# The Sustainable Development Goals and data sources for monitoring goals in Brazil

**DOI:** 10.1590/SS2237-9622202200010.especial

**Published:** 2022-08-01

**Authors:** Danielle Keylla Alencar Cruz, Aglaêr Alves da Nóbrega, Marli de Mesquita Silva Montenegro, Vinícius Oliveira de Moura Pereira

**Affiliations:** 1 Ministério da Saúde, Departamento de Análise em Saúde e Vigilância de Doenças Não Transmissíveis, Brasília, DF, Brazil

Discussions on sustainable development were included on the agenda of the United Nations (UN) in 1972, at the United Nations Conference on the Human Environment held in Stockholm, Sweden.^1^ Since then, several initiatives related to the theme, from the perspective of the construction of global agreements and agendas, marked the course of the debate between nations. Among these, Rio+20 and the Millennium Development Goals (MDGs) Agenda, which was launched in 2000 and ended in 2015, stand out.^2^

Also in 2015, a new global agenda was initiated, this time called the 2030 Agenda for Sustainable Development, adopted by 193 UN Member States. It is a global action plan, which covers the environmental, economic and social dimensions of sustainable development, in a comprehensive and interconnected manner.^3^ ^-^ ^6^ This Agenda, includes the Sustainable Development Goals (SDGs), composed of 17 goals (Figure 1) and 169 global action targets to be achieved by 2030. Guided by the global targets, the expectation is that, in addition to reaching the agreed goals, countries define their national goals and incorporate them into their policies, programs and government plans.^3^

**Figure 1:**
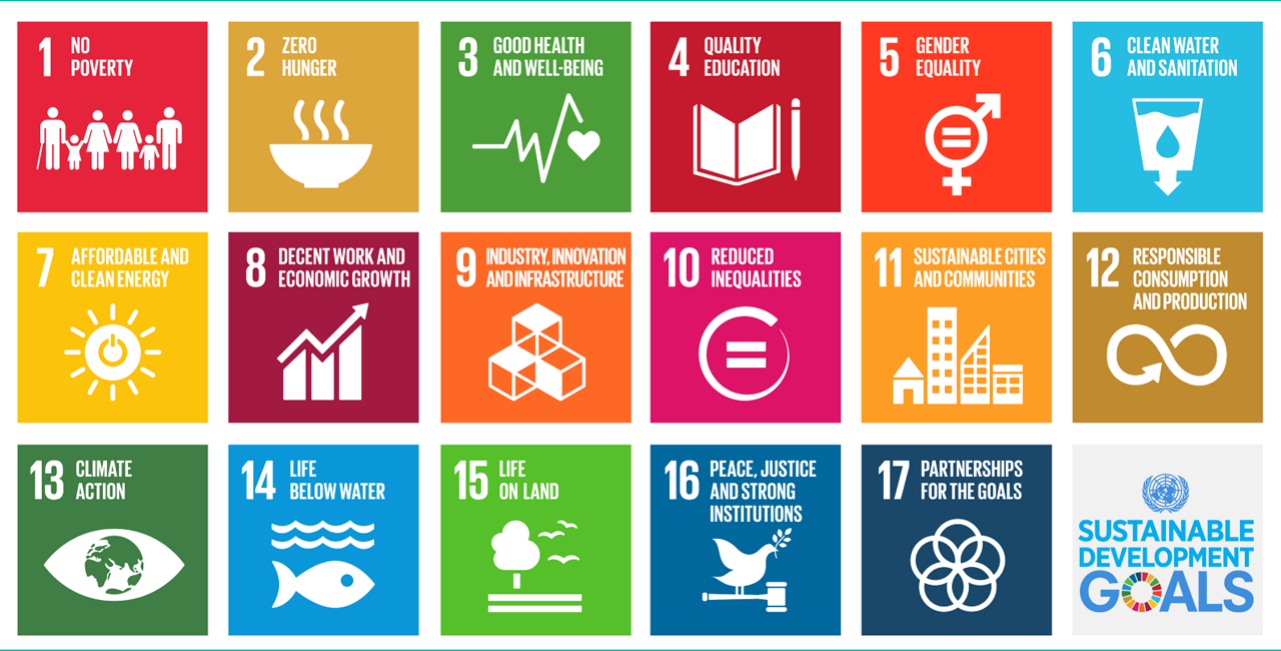
Sustainable Development Goals, 2030 Agenda

Source: United Nations Guidelines for the use of the SDG logo including the colour wheel, and 17 icons [Internet], 2019.

In this context, this paper presents some reflections on the potential of the data sources available for monitoring the goals of the SDGs in Brazil, especially the indicators used by the Health Surveillance Secretariat of the Ministry of Health (SVS/MS).

## Sustainable Development Goals in the Ministry of Health

Brazil, as a Member State of the UN, adhered to the SDGs from the moment it came into effect. In 2016, the National Commission for the SDGs (CNODS)^7^ was created with the aim of integrating, disseminating and providing transparency to the process of implementation of the UN 2030 Agenda for Sustainable Development.^7^ ^,^ ^8^ Since the revocation of that Commission in 2019,^9^ the implementation of the 2030 Agenda has been under the coordination of the Special Secretariat for Social Articulation of the Government Secretariat of the Presidency of the Republic. In the Ministry of Health, the Department of Monitoring and Evaluation of the Brazilian National Health System (DEMAS), an instance of the Ministry of Health Executive Secretariat, coordinates and articulates the monitoring and evaluation of the 2030 Agenda, and is the focal point of articulation with external institutions, with the execution of actions distributed among the departments that have goals to be monitored, due to their scope of action.

In the context of the SDGs, in addition to the political component, it is necessary to articulate initiatives in terms of monitoring and shared governance, which encourage the active participation of the entities of the federation, the civil society and the private sector in monitoring the goals, including technical support, and the implementation of local, regional and national initiatives.

In this sense, a structure was created in the Ministry of Health to organize the process of monitoring the SDGs. In 2020, a working group (WG) was created at the SVS, called GT ODS SVS, composed of representatives from all departments of the Secretariat, to work specifically on the indicators under its direct responsibility (SDG 3 - Health and Well-being), as well as those related to the themes of that secretariat (SDGs 5, 6, 8, and 16). This WG contributed so that the SDGs monitoring activities, already in place in the Ministry of Health, could be better organized and qualified. Since its implementation, the WG develops permanent activities in the following areas:

- updating of indicators (methodological form and historical data series);
- qualification of indicators that are in the 'analysis/construction' phase^3^ with the aim of updating them to the 'produced' status;^3^
- monitoring of indicators; and
- the creation of networks encompassing the Federal District, state, and municipal governments, universities and the civil society for the incorporation of the 2030 Agenda.

Faced with the need to expand the monitoring of the SDGs, in order to encompass goals and indicators related to the SDGs throughout the Ministry of Health, in 2021, through the Advisory Committee for Monitoring and Evaluation of the Brazilian National Health System (SUS), coordinated by DEMAS, the WG for the 2030 Agenda in the Ministry of Health (GT ODS MS) was created, composed of the its secretariats - the Specialized Health Care Secretariat, Primary Health Care Secretariat, the Secretariat of Science, Technology, Innovation and Strategic Health Inputs, the Special Secretariat of Indigenous Health, the Work and Education Management in Health Secretariat, and the Health Surveillance Secretariat -, the National Health Surveillance Agency, the National Health Foundation, and the National Cancer Institute.

## Monitoring the Sustainable Development Goals and the data sources from the Ministry of Health

The availability of quality, accessible, updated, reliable and disaggregated data, based on official national sources, is essential condition for the periodic production of indicators.^3^

In the Brazilian context, the following are pointed out as important data sources for monitoring the SDGs, in particular SDG 3: the Mortality Information System (SIM), the Live Birth Information System (SINASC), the Information System for Notifiable Diseases (SINAN), and the national health epidemiological surveys, such as the Surveillance System for Risk and Protective Factors for Chronic Diseases by Telephone Survey (VIGITEL), the National School-based Health Survey (PeNSE) and the National Health Survey (PNS).

The SIM is the official death registration system in Brazil^10^ and, since its creation in 1975, it has progressively improved data quality in terms of coverage, regularity and proportion of ill-defined causes.^11^ ^,^ ^12^ In 2019, a 96.2%, coverage was achieved, ranging from 89.2% to 100% among the Units of the Federation.^13^ The SINASC was created in 1990 and, in 2019, its coverage was 97.8%, ranging from 91.4% to 100% depending on the Unit of the Federation.^13^ The high coverage presented by SIM and SINASC turn these systems into robust sources of data for the calculation of indicators. SINAN, which is fed through the notification and investigation of cases of diseases and conditions from the national list of notifiable diseases,^14^ ^,^ ^15^ assists in the health sector planning and definition of intervention priorities, also enabling evaluations of the impact of such interventions, ^14^ thus demonstrating its important role in the production of indicators.

As they are official data recording systems and due to their scope, SIM, SINASC and SINAN make up the basis for calculating at least 15 of the indicators referring to SDG 3, consisting of 13 targets and 28 indicators, in addition to their use for the indicator 16.1.1, pertaining to SDG 16 (Box 1).

**Box 1:** Indicators of the Sustainable Development Goals (SDGs) calculated in the scope of the Health Surveillance Secretariat (SVS/MS), their respective data sources and classification in the Brazilian SDGs Platform

Indicators		Data sources	Classification of the indicators in the Brazilian SDGs Platform^a^
3.7.2	Adolescent birth rate (aged 10 to 14 years; aged 15 to 19 years) per 1,000 women in that age group	Live Birth Information System (SINASC); Projection and Back Projection of the Population in Brazil by sex and age group	Produced
3.1.2	Proportion of births attended by skilled health personnel	SINASC	Produced
3.1.1	Maternal mortality ratio	Mortality Information System (SIM); SINASC	Produced
3.2.1	Under-5 mortality rate		
3.2.2	Neonatal mortality rate		
3.4.2	Suicide mortality rate	SIM; Projection and Back Projection of the Population in Brazil by sex and age group	Produced
3.6.1	Death rate due to road traffic injuries		
3.9.2	Mortality rate attributed to unsafe water, unsafe sanitation and lack of hygiene		
3.9.3	Mortality rate attributed to unintentional poisoning		
16.1.1	Number of victims of intentional homicide per 100,000 inhabitants, by sex and age		
3.4.1	Mortality rate attributed to cardiovascular disease, cancer, diabetes or chronic respiratory disease		Under analysis/construction
3.3.5	Number of people requiring interventions against neglected tropical diseases (NTDs)	SINAN (Information System for Notifiable Diseases); Information System of the Schistosomiasis Control Program (SISPCE); Epidemiological Surveillance of the State of Pernambuco Secretariat of Health; Brazilian Health Information System for Indigenous Peoples (SIASI); Database of the National Survey on Schistosomiasis mansoni and Geohelminthiasis (INPEG); Website of the National Institute of Educational Studies and Research Anísio Teixeira (INEP) - Primary Education Synopsis, 2019; the Laboratory Environment Management System (GAL)	Under analysis/construction
3.3.2	Tuberculosis incidence per 100,000 inhabitants	SINAN; Projection and Back Projection of the Population in Brazil by sex and age group	Produced
3.3.4	Hepatitis B incidence per 100,000 inhabitants		Under analysis/construction
3.3.3	Malaria incidence per 1,000 inhabitants	SINAN; the Information System of the Epidemiological Surveillance of Malaria (SIVEP-MALARIA); Projection and Back Projection of the Population in Brazil by sex and age group	Under analysis/construction
3.5.2	Alcohol per capita consumption (aged 15 years and older) within a calendar year in litres of pure alcohol	Annual Industrial Annual Survey - Product (PIA-PRODUCT); Comex STAT, for alcoholic beverages Import (re-import) and Export; UN tourism statistics for alcohol consumption among tourists; the World Health Organization (WHO) statistics for unrecorded alcohol consumption; Projection and Back Projection of the Population in Brazil by sex and age group	Under analysis/construction
3.a.1	Age-standardized prevalence of current tobacco use among persons aged 15 years and older	National Health Survey (PNS); Projection of the Population in Brazil by sex and age	Under analysis/construction
3.b.1	Proportion of the target population covered by all vaccines included in their national programme	Information System of the National Program of Immunization (SI-PNI); SINASC; Back Projection of the Population in Brazil by sex and age group	Under analysis/construction
3.3.1	Number of new HIV infections per 1,000 inhabitants, by sex, age and key populations	Being defined	Under analysis/construction
3.9.1	Mortality rate attributed to household and ambient air pollution		
3.d.1	International Health Regulations (IHR) capacity and health emergency preparedness		

a) Classification on the Brazilian SDGs Platform on 11/08/21.

In terms of national surveys as sources of data for the SDG indicators, it is important to highlight that in the 2019 edition of the PNS there was an important change in relation to the previous version, conducted in 2013. In this latest edition, the minimum age of the selected resident to answer the survey questions was reduced from 18 to 15. This change occurred due to the monitoring of internationally agreed indicators, especially those related to the SDGs. To demonstrate the importance of such compliance, the SDG 3.a.1 indicator assesses the prevalence of smokers in the population aged 15 years and over.^16^ ^,^ ^17^ Thus, with the instituted change, the PNS became the data source for the calculation of this indicator. In addition to this, indicators 3.3.5 and 3.5.2 are also calculated using data from national surveys (Box 1).

Broad dissemination of the produced results is part of this monitoring process. In Brazil, this disclosure is made through the Sustainable Development Goals Digital Platform (ODS Platform),^3^ developed and managed by the Brazilian Institute of Geography and Statistics (IBGE). IBGE, Brazil's representative at the Inter-agency and Expert Group on SDG Indicators,^18^ is a contributor to this process, especially in terms of monitoring and qualifying indicators. Through integrated meetings, the internal debate within the Ministry of Health and with IBGE is strengthened in the process of designing national indicators based on the UN metadata sheets,^17^ which also supports IBGE's dialogue with the organization. The product of this articulation can be viewed on the ODS Platform,^3^ which presents the indicators to Brazil and the world, with the respective methodological sheets and historical data series, in addition to several possibilities for extracting and disaggregating information.

However, in order to monitor and achieve the goals of the SDGs, it is necessary to have intersectoral initiatives for the construction and management of networks that involve the entire Brazilian society and contribute to the formulation of policies, programs and plans. With the end of the period of validity of the 2011-2022 Strategic Action Plan to Tackle Non-Communicable Diseases in Brazil,^19^ and in response to the global agreement to reach the goals of the SDGs, the Ministry of Health developed the 2021-2030 Strategic Action Plan to Tackle Non-Communicable Diseases in Brazil, based on the contributions from its secretariats, the Federal District, states and municipalities, the private sector and the civil society. This plan, besides being updated for the goals of tackling Non-Communicable Diseases (NCDs), was expanded to include violence and accidents, health promotion and actions related to the SDGs, in addition to aligning its validity with the period of the 2030 Agenda.^20^

## Final considerations

The production of the SDG indicators is still a major challenge for the country, both in terms of quantity and diversity. Although Brazil has solid data sources, of good quality and with an adequate level of disaggregation, the 2030 Agenda requires combinations of information beyond this sector, which increases the difficulty of the process. The dispersion and lack of regularity in the production of some type of data can be cited as central issues to be observed for the production of such indicators and, consequently, the monitoring of the goals.

Lastly, it is opportune to develop the objectives and goals of the SDGs in line with the demands of the Brazilian National Health System (SUS). This way, possibilities are created for its reach and the strengthening of intersectorality, universalization and equity in health, requirements to contemplate the diversity and complexity of the themes of the 2030 Agenda in face of the social, political, economic, cultural and environmental health determinants. 
